# Healthy Hospitals

**Published:** 1888-05-05

**Authors:** 


### HEALTHY HOSPITALS.

The valuable and interesting paper lately read before the Royal Institute of British Architects by Mr. Vivian B. Lewes, of the Royal Naval College, opens out a new vista of hope in the minds of those sanitarians who have for years felt that the sources from which we obtain artificial light are also sources which vitiate the atmosphere and affect the health. A debt of gratitude is due to him not only for showing how these ill effects may be prevented, but also for impressing upon us the extent of the mischief at present existing. Most people are aware that candles, lamps, or gas burning in a room, produce a feeling of oppression; but it has been left to Mr. Lewes to point out the amount of the vitiation of the air which takes place; and he has done this in the most practical way by showing the number of people in a room who would give off during respiration the same amount of carbonic acid gas as each of our more ordinary illuminants. It is startling to find that three sperm candles of the size known as "sixes" are equal to two men, and that an ordinary oil lamp burning 1,980 grains of oil per hour does as much in fouling the air as fourteen people. Speaking of gas, Mr. Lewes points out that the amount consumed and the consequent impurities sent into the air, depend upon the kind of burner used. It is satisfactory to know that striking improvements have been made in this direction quite recently. The Wenham Company's lamp has been proved to be the best hitherto introduced to the public. This lamp, with a consumption of 3·2 cubic feet of gas per hour will give as much light as the oil lamp burning its 1,980 grains of petroleum, whilst the amount of vitiation is only equal to 2·6 adults instead of to 14. This of course is a highly important point, but still more important is the fact that these lamps can be made most powerful engines for ventilation. The Wenham lamp is now extensively used as a ventilating light, and, being an overhead lamp, can be easily fitted into an ornamental ventilating shaft connected with a flue, and under these conditions it has been found by experiment that it removes from 700 to 2,000 cubic feet of air from the upper part of the room, besides all its own products of combustion, the volume of air removed depending upon the quantity of gas consumed.

The point we wish to draw special attention to is the important bearing these facts have upon the illumination and the purification of the air of hospital wards. In our hospitals perfect ventilation is at the same time one of the most important and most difficult problems which have to be solved, and anything which helps towards the desired result must be of enormous value. In illness and convalescence patients are more sensitive to any trace of pollution in the air breathed than at any other time, and thousands of cases might be adduced in which the trembling scale has been turned in the wrong direction; and, indeed, in which serious epidemics have arisen from faulty ventilation. This consideration has always made the subject of lighting hospital wards one of special difficulty; but with this new departure in the method of burning coal gas the difficulty seems to be immensely diminished. The general adoption of the Wenham ventilating lamps for this purpose would be a marked advance in the right direction.

Another point which must not be overlooked is that these lamps do not heat the air in the same way that an ordinary flame does. All the heat emitted by them when used in their ventilating form is radiant heat, which is undoubtedly the least oppressive and most healthful way in which heat can be imparted to a room or ward, and from the height at which they are fixed even this is imperceptible. The thanks of the community at large, and of hospital managers especially, are due to Mr. Lewes for this paper, in which he has drawn public attention to a highly important aspect of hospital and general sanitation.

